# Immunogenicity and protective efficacy of the recombinant *Pasteurella multocida* lipoproteins VacJ and PlpE, and outer membrane protein H from P. multocida A:1 in ducks

**DOI:** 10.3389/fimmu.2022.985993

**Published:** 2022-10-07

**Authors:** Yajuan Li, Junfang Xiao, Yung-Fu Chang, Hui Zhang, Yutao Teng, Wencheng Lin, Hongxin Li, Weiguo Chen, Xinheng Zhang, Qingmei Xie

**Affiliations:** ^1^ Heyuan Branch, Guangdong Provincial Laboratory of Lingnan Modern Agricultural Science and Technology, College of Animal Science, South China Agricultural University, Guangzhou, China; ^2^ Guangdong Engineering Research Center for Vector Vaccine of Animal Virus, College of Animal Science, South China Agricultural University, Guangzhou, China; ^3^ College of Veterinary Medicine, Cornell University, Ithaca, NY, United States; ^4^ South China Collaborative Innovation Center for Poultry Disease Control and Product Safety, College of Animal Science, South China Agricultural University, Guangzhou, China

**Keywords:** *Pasteurella multocida*, outer membrane protein, lipoprotein, immunogenicity, protective efficacy

## Abstract

Duck cholera (duck hemorrhagic septicemia) is a highly contagious disease caused by *Pasteurella multocida*, and is one of the major bacterial diseases currently affecting the duck industry. Type A is the predominant pathogenic serotype. In this study, the genes encoding the lipoproteins VacJ, PlpE, and the outer membrane protein OmpH of *P. multocida* strain PMWSG-4 were cloned and expressed as proteins in *E. coli*. The recombinant VacJ (84.4 kDa), PlpE (94.8 kDa), and OmpH (96.7 kDa) proteins were purified, and subunit vaccines were formulated with a single water-in-oil adjuvant, while killed vaccines were prepared using a single oil-coated adjuvant. Antibody responses in ducks vaccinated with recombinant VacJ, PlpE, and OmpH proteins formulated with adjuvants were significantly antigenic (p<0.005). Protectivity of the vaccines was evaluated *via* the intraperitoneal challenge of ducks with 20 LD50 doses of *P. multocida* A: 1. The vaccine formulation consisting of rVacJ, rPlpE, rOmpH, and adjuvant provided 33.3%, 83.33%, and 83.33% protection, respectively, the vaccine formulation consisting of three recombinant proteins, rVacJ, rPlpE, rOmpH and adjuvant, was 100% protective, and the killed vaccine was 50% protective. In addition, it was shown through histopathological examination and tissue bacterial load detection that all vaccines could reduce tissue damage and bacterial colonization to varying (p<0.001). These findings indicated that recombinant PlpE or OmpH fusion proteins formulated with oil adjuvants have the potential to be used as vaccine candidates against duck cholera subunits.

## Introduction

*P. multocida* is a typical commensal of the upper respiratory tract (1, 2) and can cause various animal diseases such as swine atrophic rhinitis and swine pneumonia, and hemorrhagic septicemia in various domestic poultry (3). The disease of ducks caused by *P. multocida* is called duck cholera or duck hemorrhagic septicemia, an acute, highly transmissible, and septicaemic infection with an incidence of 30-70% and a mortality rate of 30%-80% (4, 5). Duck cholera is one of the major bacterial diseases currently affecting the duck farming industry. Some data indicate that approximately 30% of poultry die each year worldwide due to avian cholera, causing enormous economic losses to the poultry farming industry worldwide (6, 7). Previously, antibiotics were mainly used for the prevention and treatment of bacterial diseases, but large scale antibiotic usage led to the selection of many bacterial resistance markers (4, 8). Currently, vaccination is the standard measure aimed at disease prevention. The commercial vaccines against *P. multocida* are live attenuated and killed vaccines (9, 10). Still, both vaccines have drawbacks, such as inducing short-term protection, producing weak immunity, and possibly regaining virulence (9, 11). Considering the limitations of current vaccines, the development of safe and effective subunit vaccines is necessary (12). As an approach to ameliorate the efficacy of the commercially applied killed vaccines and to enhance the safety of attenuated vaccines, the use of combined regimens of killed and attenuated vaccines were evaluated (13). The new regimen showed promising results, especially when formulated with a safe natural product such as propolis (14). The commercial success in using whole cell vaccines, does not block the way for continuing trials to apply protein subunit vaccines in the veterinary field. Protein subunits have the advantage of being expressed on bacterial ghost platforms (15). Together with acting as cheap production tool for the target proteins, ghost-platforms are powerful carriers of the immunogens to the immune cells with no need for using adjuvants.

Outer membrane proteins and lipoproteins are play key roles in the interaction of the pathogen with the host environment and in the host immune response to infection, they function as enzymes, inhibitors, transporters, structural proteins, as virulence factors and activate the innate immune system (12, 16). Recent studies have demonstrated that vaccination of several outer membrane proteins (OMPs) of *P. multocida* could induce immunogenic responses with a bactericidal activity of the immune sera (17, 18). Among them, the virulence-associated chromosome locus J (*vacJ*), a widely distributed and highly conserved outer membrane protein gene that plays a virulence-associated role in most Gram-negative bacteria and was first identified in *Shigella flexneri* as a gene associated with bacterial transmission (19). The PM1501 gene of a *P. multocida* isolate (Pm70) is predicted to be a homolog of VacJ and encodes a protein of about 27.5 kDa (18), which elicits a humoral immune response with a significant increase in antigen-specific IgG titers and has emerged as a new target for the induction of protective immune responses in mice (20), their study established the immunogenicity and protective efficacy of lipoprotein VacJ. The outer membrane protein H (OmpH) is approximately 33.8 kDa, and is one of the major outer membrane proteins associated with the pore protein family in *P. multocida* (21); it is present in a variety of serotypes of *P. multocida*, present in the outer membrane as a homotrimer (22). It has been shown that OmpH has the potential to be an immunogenic protective antigen (23–27). The native OmpH was shown to be able to induce protective immunity in chickens against homologous strain challenge (28). Tan etal. (29) demonstrated that rOmpH vaccination could cause a high humoral response, recombinant OmpH vaccine was safe and effective. *P. multocida* lipoprotein E (PlpE), with a molecular weight of about 38 kDa, is an important immunogenic outer membrane protein in *P. multocida* (30); According to the found of Mostaan etal. (31), PlpE had immunogenicity, antigenicity, different serotypes coverage, and antibody accessibility, it was a lipid-modified surface-exposed outer membrane protein with an important role in complement-mediated killing (32), and previous studies demonstrated that recombinant PlpE is protective and safety in mice, rabbits, chickens and calves (30). The PlpE gene is widely present and has a high homology among serotypes of *P. multocida* (32).

This study aimed to investigate the immunological efficacy of recombinant VacJ, PlpE, and OmpH proteins from *P. multocida* type A: 1 in the duck model. The recombinant proteins were emulsified with a single oil-packed adjuvant and inoculated into ducks. The protective effect was assessed by the survival rate of ducks under lethal infectious doses. Our study showed that the protection against the challenge was 33.33%, 83.33%, 83.33%, 100% and 50% in the rVacJ, rPlpE, rOmpH, rVacJ+rPlpE+rOmpH and killed vaccine groups, respectively. These results indicated that the purified duck *P. multocida* outer membrane proteins PlpE and OmpH could induce strong immunogenicity, but not VacJ. However, the combination of these three proteins resulted in enhanced immunogenicity and better protection of the vaccinated ducks against a challenge with a virulent strain.

## Materials and methods

### Bacteria, vector, and test animals

*P. multocida* group A: 1 strain PMWSG-4 was isolated from the liver of a duck and preserved for our laboratory. The pET43.1a vector, *E. coli* DH5α, and BL21 (DE3) cells were used to construct expression clones. The required primers (**Table S1**) were synthesized and obtained (Biobiotics Shanghai Ltd). One-day-old ducklings were purchased from the Guangdong Wenshi Duck Factory and used for a vaccine trial.

### The construction of recombinant plasmids

The protein sequences of the target genes VacJ (GenBank accession no. AAK03585.1), PlpE (GenBank accession no. ABP93661.1), and OmpH (GenBank accession no. AAC02243.1) were obtained from GenBank and analyzed by SignalP, the signal peptide was removed, and the His tag was added. Primers (**Table S1**) were designed to amplify target fragments using the principle of homologous recombination. Strain PMWSG-4 chromosomal DNA was extracted using the omega bacterial genome kit (Omega USA) and used as a template to amplify the VacJ, PlpE, and OmpH genes. Specifically, the PCR mixture contained 23ul autoclaved distilled water, 25ul 2× PrimeSTAR Mix (Vazyme Biotech), 1ul DNA Template, and 1ul of each forward and reverse primer (working concentration: 10 umol/L). Each of the 30 PCR cycles consisted of 95°C for 5 min, 94°C for 30 s, 60°C for 30 s, 72°C for 1 min, and ending with 72°C for 5 min. The purified PCR amplification product was ligated to the pET43.1a vector using a homologous recombination kit (NCM Biotech). Transformant cells were streaked on LB plates with ampicillin (50 mg/ml), and the recombinant plasmids were verified by nucleotide sequence analysis.

### Purification of recombinant proteins and immunoblotting

*E. coli* BL21 cells carrying pET43.1a-VacJ, pET43.1a-PlpE, and pET43.1a-OmpH were grown in Luria Broth (LB) supplemented with 50mg of ampicillin/ml. Expression was induced at OD600 of 0.6 by adding isopropyl-b-D-galactopyranoside (IPTG) to 1 mM final concentration and followed with culturing at 37°C for 12 h in a shaker incubator at 150 RPM. Cells were collected by centrifugation and resuspended in PBS buffer (8.0 g NaCl, 0.2 g KCl, 1.44 g Na2HPO4, 0.24 g KH2PO, pH 7.4). Pellets were fragmented by ultrasonication (300W power, crushing 4S, 6S interval, a total of 30 minutes). The lysate was spun at 12,000 rpm for 30 min, and the supernatant was harvested. Proteins were purified by nickel affinity chromatography using Ni–NTA Beadose Resin kit (Jiangsu Cowin Biotech Co., Ltd.) as per the standard manufacturer’s protocol (33), following column binding and washing with buffer (PBS, pH 7.4, 50 mM Imidazole), and proteins were eluted using PBS buffer (containing 250 mM imidazole).

The purity of the recombinant protein was determined by SDS-PAGE. Western blot analysis was carried out using the Anti-His-Tag Mouse Monoclonal Antibody as the primary antibody of 1:1000, CoraLite488-conjugated Affinipure Goat Anti-Mouse IgG (H+L) was used as a secondary antibody at a dilution of 1: 10,000. Colometric detection was performed using an ECL substrate luminescence kit.

### Vaccine formulations

Preparation of killed bacterins: revived strain PMWSG-4 was grown; 1 mL was taken for plate counting. After counting, the bacterin was adjusted to a standard density (8×10^8^ CFU/ml), and formaldehyde was added at a final concentration of 0.5%. Bacterin was incubated at 180 rpm and 37°C for 24 hours. After inactivation, formaldehyde killing of *P. multocida* - was confirmed by plating in a tryptic soy agar plate and incubated in a 37°C incubator overnight. A single-phase water-in-oil adjuvant (provided by Guangdong Wen’s Foodstuff Group Co. Ltd) was added to the inactivated vaccine and emulsified into “water-in-oil” by an emulsifier stored at 4°C.

Preparation of subunit vaccines: Recombinant protein samples of known concentrations were mixed with single-phase water-in-oil adjuvant to ensure 100µg of purified protein per 500 µl of vaccine emulsion (the ratio of protein to adjuvant is 2:3). The recombinant vaccine preparations were further emulsified (9,000 rpm, 10min, 4°C) by homogenizer (IKA Germany) to ensure homogeneity and stored at 4°C.

### Vaccination in a duckling model

In animal experiments, 10-day-old ducks were used, divided into 7 experimental groups with 15 animals per group. Vaccines were delivered subcutaneously in the neck on days 14 and 28, each duck in a Group 2、Group 3、Group 4 and Group 5 were inoculated with rVacJ protein (100μg/dose)、rPlpE protein (100μg/dose) 、rOmpH protein (100μg/dose) and rVacJ/rOmpH/rPlpE proteins (100µg each/dose) in a 0.5ml volume mixed with adjuvant. Each duck in a Group 1 and Group 7 were inoculated with PBS and adjuvant in 0.5ml. The PBS group was blank control group. twenty-eight days after the booster, Ducks in Group 2-7 were challenged with a PMWSG-4 strain with 20 LD50 (unpublished) in the leg muscle (the PMWSG-4 strain has been previously studied with an LD50 of 4.5 CFU).

Clinical signs, appetite, and mortality were observed daily and recorded for 14 days post-challenge. Dead ducks were dissected, and liver tissues were aseptically collected for bacterial isolation. In addition, blood was collected from the neck at 14, 28, and 42 days dpi for serum antibody testing. The animal experiments were approved by the Animal Experimentation Ethics Committee of South China Agricultural University (Approval number of Ethics Committee:2021b184). The protective effects were determined by the survival time of the ducks within 14 days post-challenge. According to a previous method (34), the mortality of the challenged ducks was expressed as percentage survival and mean survival time (MST), plotted percentage survival curve.

Examination of the histopathological lesions in ducks was carried out as previously described (19, 34). At 24 h post-challenge, three randomly selected ducks from each group were selected and euthanized; hearts, livers, spleens, lungs, and kidneys were harvested and fixed in 4% paraformaldehyde, embedded, sectioned, and subjected to (HE) staining for histopathological examination. In addition, bacterial loads were measured by real-time qPCR tests performed on DNA preparations made from the extracted heart, liver, spleen, lung, and kidney tissues. Specifically, primers(F:5’-TAACGGCAGAGCGGTTTAAT-3’,R:5’-GCTGTAAACGAACTCGCCA-3’) were designed according to the KMT1 gene (35) of *P. multocida*; HiScript II One Step RT-PCR Kit (Vazyme Biotech) was used as real-time qPCR, and the qPCR mixture contained 7.2 ul autoclaved distilled water, 10μl 2 × One Step SYBR Green Mix, 1μl One Step SYBR Green Enzyme Mix, 1μl RNA Template, 0.4μl of forward and reverse primer each (working concentration: 10 μmol/L), the amplification program was 50°C for 15 min, then each of the 40 qPCR cycles consisted of 95°C for 30 s, 95°C for 10 s, 60°C for 30 s, finally 95°C for 15 s, 60°C for 60 s, 95°C for 15 s.

### Enzyme-linked immunosorbent assay

Antibody titers were measured by indirect ELISA, as previously indicated (36). In addition, specific antibody responses were measured by measuring IgG titers *via* ELISA using sera collected from vaccinated ducks. Briefly, purified recombinant VacJ, PlpE, and OmpH proteins were used as coating antigens at concentrations of 3 μg/mL, 2.5 μg/mL, and 2 μg/mL for the determination of anti-VacJ, anti-PlpE, and anti-OmpH antibody levels, respectively. Dilutions of the duck sera ranging from 1:500 to 1:16,000 were used in triplicates as primary antibodies; antibody to duck IgG conjugated to alkaline phosphatase (KPL, USA) was used as a secondary antibody at a dilution of 1:1,000. TMB Chromogen Solution (Sangon Biotech, China) was used as the color development reagent. Plates were read at 405 nm to determine optical density on a microtiter plate reader.

### Statistical analysis

The data are shown as the means ± SD. An analysis of variance (ANOVA) was used for the mean comparison of antibody response between groups. All statistical analyses were performed using GraphPad (GraphPad Software, California, USA). Differences were considered statistically significant at p<0.05.

## Results

### Cloning and purification VacJ, PlpE, and OmpH recombinant proteins

**Figure 1:**
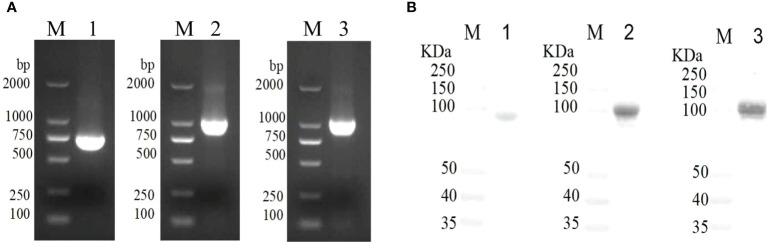
Gene cloning and immunogenicity of rVacJ, rPlpE,rOmpH lipoprotein in a duck model. Panel **(A)** PCR amplification of VacJ, PlpE, OmpH genes from *P. multocida* A:1 strain PMWSG-4. Lane M: DNA standard ladder, Lane 1,2 and 3: Amplified VacJ(746bp), PlpE(1021bp), and OmpH(1083bp) gene product. Panel **(B)** Western blot of protein rVacJ, rPlpE and rOmpH. Protein standard marker (lane M), Immunoblot of rVacJ(84.4kDa), rPlpE(94.8kDa), rOmpH(96.7kDa) (lane 1, 2 and 3).

The VacJ (746bp), PlpE (1021bp), and OmpH (1083bp) genes were cloned from the genomic DNA of *P. multocida* PMWSG-4 by PCR, and the genes were validated by DNA sequences (**Figure 1A**). VacJ, PlpE, and OmpH were cloned into pET43.1a to express his-tagged fusion proteins. The recombinant plasmids were identified by DNA sequencing, indicating that the target genes were accurately cloned into pET-43.1a between the SmaI and HindIII sites. The purity of the recombinant proteins was checked on SDS-polyacrylamide gels by Komas Brilliant Blue staining and proved by the Western blot analysis. Purified recombinant VacJ, PlpE, and OmpH proteins showed bands on SDS-polyacrylamide gels with expected molecular weights of 84.4 kDa, 94.8 kDa, and 96.7 kDa, respectively (**Figure 1B**). Western blot analysis using specific antibodies showed that these antibodies could react antigenically with the purified recombinant proteins.

### Humoral responses to VacJ, PlpE, OmpH protein, and killed vaccines and their protective effects

**Figure 2:**
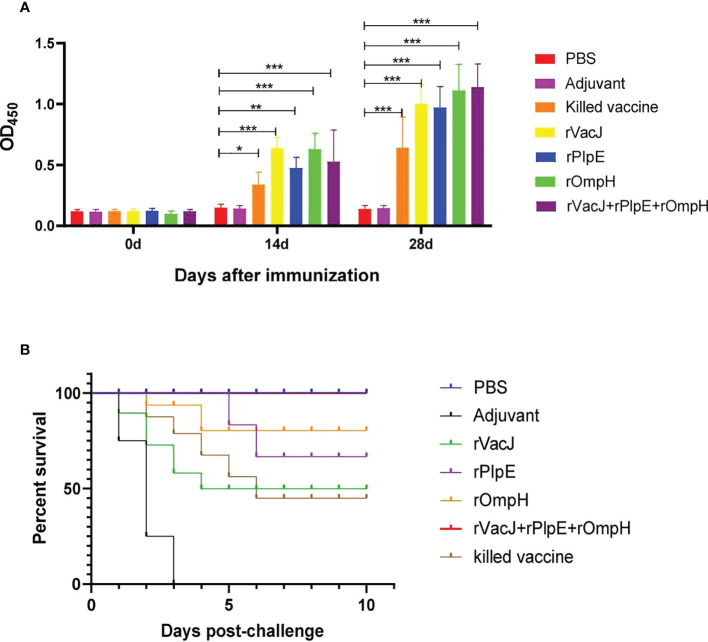
In a duck model, the ELISA and protective efficacy of rVacJ, rPlpE, and rOmpH proteins. Panel **(A)** The bar diagram indicating the rVacJ, PlpE, and OmpH antibody titers (total IgG) of sera collected from all the ducks at 0, 14, and 28 days post-immunization. Panel **(B)** The survival/mortality rates of vaccinated and control ducks (12 ducks/group) following challenge with 20 LD50 of *P. multocida* A:1 strain PMWSG-4. *: P<0.05, **: P<0.01, ***:P<0.001.

The ducks were inoculated twice with rVacJ, rPlpE, rOmpH, rVacJ+rPlpE+rOmpH protein and killed bacterin. The sera were collected prior to booster and challenge (28 and 42 days, respectively) and used to determine ELISA titers. After the first immunization, serum IgG levels were significantly elevated in the rVacJ, rPlpE, rOmpH and rVacJ+rPlpE+rOmpH groups, which differed considerably from the control group (p<0.005). The killed vaccine group differed substantially from the adjuvant group (p<0.05). In contrast, the antibody levels in the rVacJ and rOmpH groups were higher than those in the killed vaccine group, and the difference was highly significant (p<0.001) (**Figure 2A**). After the second immunization, antibody levels in all immunized groups were higher than in the advanced group, with significant differences (p<0.0001). rOmpH and rVacJ+rPlpE+rOmpH antibody levels were higher than those in the killed vaccine group, with significant differences (p<0.005), and rVacJ and rPlpE groups were significantly different from those in the killed bacterin group (p<0.05) (**Figure 2A**).

The protective efficacy of recombinant proteins formulated with oil adjuvants was investigated after the challenge of immunized ducks with 20 LD50 doses of live *P. multocida* A: 1. The ducks in the rVacJ group showed slight depression and loss of appetite after the challenge and died from day one to day four; Only a few ducks in the rPlpE and rOmpH groups showed depression and loss of appetite, with the deaths in the rPlpE group occurring on days 5 and 6 after the challenge, and the deaths in the rOmpH group occurring on days 2 and 3, respectively; The ducks in the rVacJ+rPlpE+rOmpH group showed no any abnormalities or deaths; In the killed vaccine group, the ducks showed more severe depression and loss of appetite, and died one after another during the first to fifth days post-challenge; In the adjuvant group, ducks showed apparent depression and loss of appetite and began to die on the first-day post-challenge. All the ducks in PBS group grew normally. The mortality of the ducks in each group after the challenge is shown in (**Table 1**; **Figure 2B**). The vaccine formulation consisting of rVacJ, rPlpE, rOmpH, and adjuvant provided 33.3%, 83.33%, and 83.33% protection, respectively, and the vaccine consisted of three recombinant proteins, rVacJ, rPlpE, and rOmpH with adjuvant provided 100% protection. The killed bacterin provided 50% protection (**Table 1**). In addition, *P. multocida* was re-isolated from the post-mortem liver tissue of each duck and confirmed to be *P. multocida* A:1 by standard bacteriological tests and PCR, amplifying 460bp and 1300bp, respectively.

**Table 1:** The survival rate of ducks after *P. multocida* challenge.

Group	No. of duck survived/challenged	Death time	Protection (%)
PBS	12/12		100
rVacJ	4/12	2-4 day	33.33
rPlpE	10/12	5、6 day	83.33
rOmpH	10/12	2、3 day	83.33
rVacJ+rPlpE +rOmpH	12/12		100
Killed vaccine	6/12	2-6 day	50
Adjuvants	0/12	2-3 day	0

### Histopathological changes and viral tissue load of the ducks in each group after challenge

**Figure 3:**
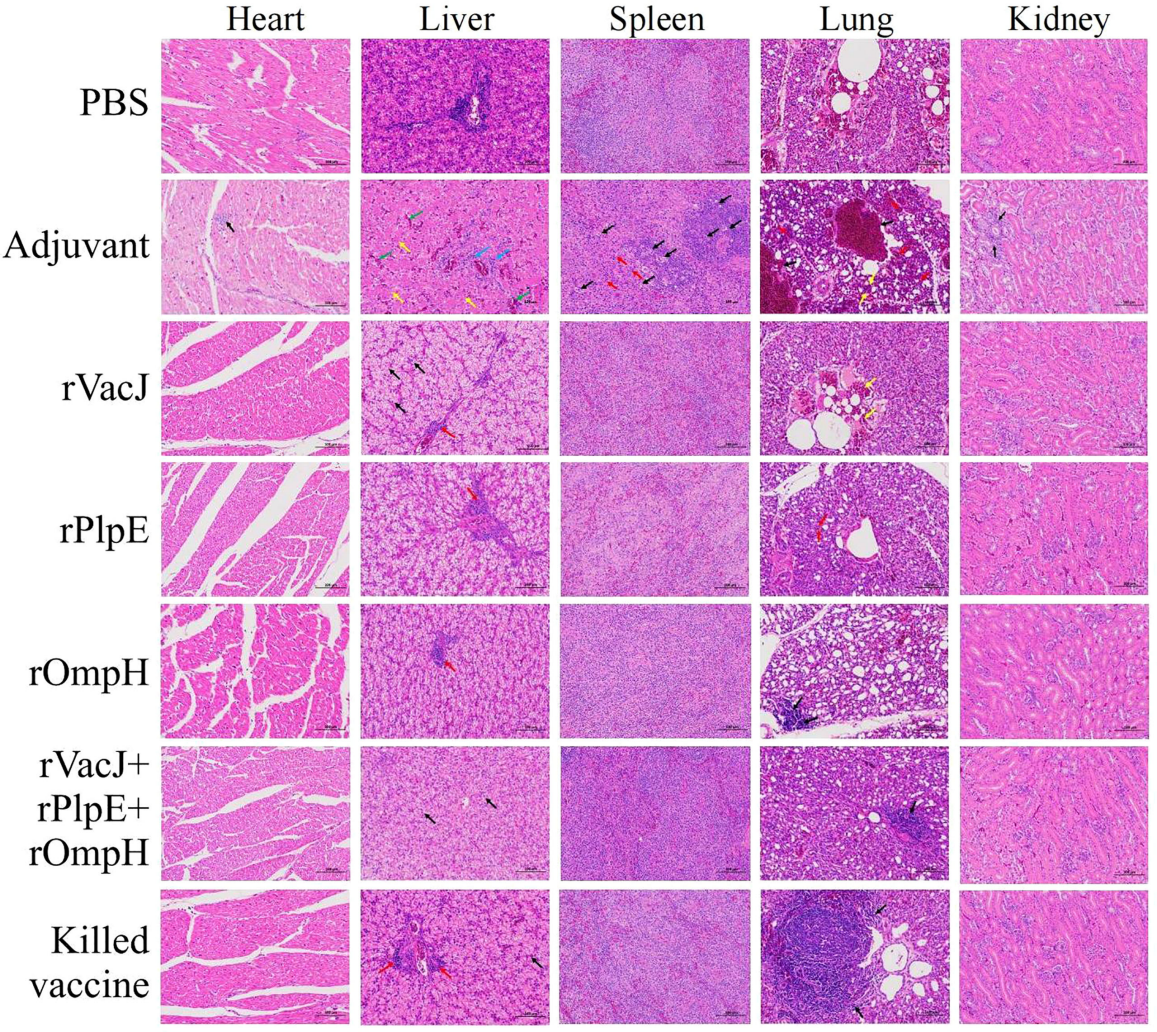
Ducks in Group 2-7 were challenged with 20-fold LD50 of the *P. multocida* PMWSG-4 strain intramuscularly 14 days after the booster. Then, histopathological lesions in the heart, liver, spleen, lung, and kidney (n=3/group) were analyzed by HE staining at 3 days post-challenge. The representative results of each group were shown.

​The histopathological analysis post-challenge shows that ducks in the adjuvant control group had severe heart, liver, spleen, lung, and kidney lesions with the structural disorder. In contrast, the immunized vaccine group showed no significant lesions in the heart, spleen, and kidneys and minor damage to the liver and lungs. Specifically, the adjuvant control group exhibited inflammatory cell infiltration in heart tissue (black arrows), and other groups displayed regular tissue structures without histopathological lesions. The adjuvant group liver tissue showed mild degrees of hepatic steatosis, and the cytoplasm of the hepatocytes appeared vacuolation (yellow arrows); there was a clear hepatic sinusoidal dilatation and congestion (green arrows), and had a slight infiltration of inflammatory cells around the vessels (blue arrows). The hepatocytes were swollen, and cytosol appeared vacuolated (black arrows), with a slight infiltration of inflammatory cells around the vessels (red arrows) in the rVacJ and killed vaccine groups. rPlpE and rOmpH vaccinated groups had a focal perivascular inflammatory cell infiltration (red arrows). rVacJ+rPlpE+rOmpH groups showed cellular swelling of the hepatocyte, and cytosol appeared vacuolated (black arrow). In the adjuvant group, spleen tissues were marked with necrosis and inflammation in the white pulp; lymphoid nodules, periarteriolar lymphocyte sheaths, and reticulocytes were necrotic (black arrows); light heterophilic granulocyte infiltration was observed (red arrows), while the spleens of other groups were normal. The lung tissue from the adjuvant group featured bronchus intraluminal bleeding and was filled with erythrocytes (black arrows); capillary congestion on respiratory surfaces (red arrows), and respiratory surface thickening (yellow arrows). rVacJ group had bronchus intraluminal bleeding filled with erythrocytes (yellow arrows); rPlpE group had respiratory surface thickening (red arrows) and decreased capillary density; other groups had intrabronchial inflammatory cell infiltration (black arrows). In kidneys, renal tubular interstitial vascular congestion was observed (black arrows) in adjuvant groups; other groups displayed no obvious tissue pathologies (**Figure 3**).

**Figure 4:**
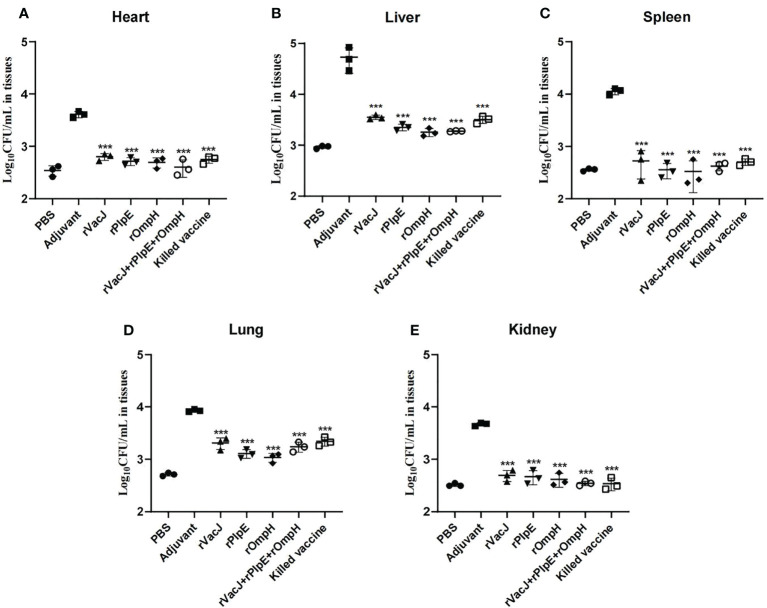
Bacterial loads in various organs are determined by qRT-PCR. The bacterial loads of each vaccination group (n=3/group) in the heart **(A)**, liver **(B)**, spleen **(C)**, lung **(D)** and kidney **(E)** were determined as CFU/mL at 24 h post-challenge. The PBS group was blank control group (not challenged with PMWSG-4 strain). *** is P<0.001.

Bacterial loads in the heart, liver, spleen, lung and kidney tissue measured by Real-time qPCR were significantly lower than those in the adjuvant control group(p<0.005) (**Figure 4**), consistent with histological lesions, suggesting that ducks delivered with recombinant protein formulas provided significant protection against challenge.

## Discussion

Bacterial transmembrane proteins and lipoproteins play a key role in the interaction of pathogens with the host (37); in recent years, researchers have identified several gene products that could be used as vaccine candidates from the *P. multocida* genome. These include the outer membrane lipoproteins VacJ, PlpE and outer membrane protein OmpH (38, 39). We found that VacJ proteins were highly conserved regardless of serogroup and host species origin (19), with homology in the range of 98.9%-99.3% (**Figure S2A**). The phylogenetic tree shows that VacJ proteins of strain PMWSG-4 and type A: 1 isolated from sheep and rabbits clustered into one branch (**Figure S1A**). The homology of the PlpE protein from type A:1 was 87.6-94.7%; interestingly, only 50% homology to that of porcine-derived D isolates (**Figure S2B**). Our results slightly differ from Wu etal. (32), they indicated 90.8-100% homology among plpE gene sequences among *P. multocida* isolates. The phylogenetic tree clusters into a single branch with the chicken-derived A: 1 type X-73 (**Figure S2B**). The homology of the OmpH protein with other serotypes ranged from 77.4% to 96.7%, and the phylogenetic tree clustered with chicken-derived A: 1 type X-73 (**Figure S1C**), with the highest homology with chicken-derived type A (**Figure S2C**). The three proteins are widely distributed among serotypes of *P. multocida*, and VacJ proteins are highly conserved; the PlpE protein of serotype A is more homologous to that of type A of the same species; The OmpH protein of serotype A is less homologous to that of OmpH proteins from serotypes B and D. It is presumed that vaccines of serotype A are likely to provide a cross-protection among serotypes.

Regarding “reducing and prohibiting antibodies”, vaccines are considered the most effective means of preventing bacterial diseases. Previously, the prevention of *P. multocida* was mainly vaccinated with live or killed vaccines (40); however, both had some drawbacks and lacked efficacy, prompting the development of new, safe, and effective subunit vaccines (39, 41, 42). Previous studies have indicated that lipoproteins VacJ, PlpE, and outer membrane protein OmpH recombinant vaccine studies against different serotypes of *P. multocida* are of interest. In our study, immunization with 100µg/duck of rVacJ, rPlpE, and rOmpH, and killed vaccine elicited a high humoral response with significantly elevated serum IgG levels (P<0.005). However, the protective effects of rVacJ, rPlpE, rOmpH protein, and killed vaccines were interesting. Compared with previous studies. Sathish et al. (20) indicated that vaccination of mice with 75µg/mouse rVacJ elicited a humoral immune response that resulted in a substantial increase in antigen-specific titers of IgG and its subtypes (IgG1 and IgG2a) and with 66.7% protection after challenge with serotype B: 2 (8LD50). In our study, under the 20 LD50 challenge, the rVacJ vaccinated group showed only a 33% protection, the protection of rVacJ is poor. However, challenged with the high lethal dose may be the reason for the low protection rate. Wu etal. (32) reported that the immunization with 100 µg/chicken for rPlpE vaccine from *P. multocida* serotype A: 1 had a 63-100% survival rate after being challenged with a lethal dosage of serotypes A: 1, A: 3, and A: 4. Sezer Okay etal. (36) indicated that vaccine formulations consisting of rPlpE and oil-based or oil-based CpGODN provided 80% and 100% protection under a 10 LD50 challenge. Hemorrhagic septicemia caused by type B: 2, Anucha et al. (43) showed that the rOmpH intranasal vaccine could induce antibody and cell mediated responses, with 83.33% and 100% protection. Tan etal. (29) demonstrated that rOmpH vaccination could obtain 80% and 100% protection against challenge with *P. multocida* serotype B: 2 intraperitoneally. In addition, the recombinant rOmpH vaccine for fowl cholera showed 90% protection in a mouse model (44). Our study was consistent with the previous studies, both rPlpE and rOmpH showed a protection rate of 83.33%. Interestingly, the recombinant rPlpE subunit vaccine induced earlier protection than the rOmpH subunit vaccine. Also, Previous studies have shown that protection rates of monovalent rVacJ, rPlpE, and rOmpH were from 60% to 100% (20, 36), And only in the case of high immune dose or low challenge dose, the protection rate could be 100%. But in our study, the rVacJ+rPlpE+rOmpH trivalent vaccine group showed a 100% protection rate. At the same time, previous studies showed that the killed vaccines against *P. multocida* have a protective effect of 50% to 100% (40, 45, 46). Our research showed the killed vaccinated group only have 50% protection, Compared to subunit vaccines, the individual immunogenic component as well as antibody level of subunit vaccine is higher than that of killed vaccine. Therefore, the protective effect of the subunit vaccine under 20LD50 challenge would be higher than that of killed vaccine. Moreover, the rVacJ+rPlpE+rOmpH trivalent vaccine group showed a 100% protection rate; our findings indicated that the trivalent vaccine was better than the monovalent recombinant subunit vaccine (44) and attenuated live vaccine (40, 47, 48).

Zhao etal. (49) developed a novel attenuated mutant *P. multocida* vaccine strain, which could reduce bacterial loads in blood and organs after oral or intranasal vaccination. In this study, histopathology results after challenge revealed that ducks in the adjuvant group showed considerable pathological changes in the heart, liver, spleen, lungs, and kidneys, while ducks in the other vaccinated groups showed insignificant pathological lesions in the liver and lungs, heart, spleen, and kidney tissues. Likewise, the Real-time qPCR results (**Figure 4**) indicated that the immunized ducks had reduced bacterial loads on tissues such as the liver and lung, and no bacteria were detected in the immunized group’s heart, spleen, and kidney tissues. Compared with the PBS group, the bacterial load of the heart, liver, spleen, and kidney tissues in the vaccine group was not significantly different (ns). However, there were significant differences in lung tissue between PBS group and rVacJ and killed vaccine group (P <0.05),this may be one of the reasons for the low protection rate of rVacJ and killed vaccine. This result shows that both the subunit vaccine and killed bacterin could reduce the histopathologic lesions.

In conclusion, the present study was the first to combine rVacJ, rPlpE and rOmpH antigens to formulate a multivalent vaccine and showed a 100% protection rate after challenge with type A: 1 *P. multocida* isolated from ducks. The monovalent rPlpE and rOmpH vaccines had an 83.3% protection rate. Subcutaneous vaccination of the neck induces high levels of serum IgG, decreases the bacterial loads in the heart, spleen, liver, lungs, and kidneys, and reduces damage to the spleen, liver, and lungs.

## Data availability statement

The original contributions presented in the study are included in the article/**Supplementary Material**. Further inquiries can be directed to the corresponding author.

## Ethics statement

The animal experiments were approved by the Animal Experimentation Ethics Committee of South China Agricultural University.

## Author contributions

QX obtained funding. YL and JX designed a trial protocol. YT and WL completed the construction of recombinant plasmid and protein purification. HL prepared the vaccine. YL and JX completed the duck immune evaluation test. HZ and YC were performed to observe histopathological changes by eosin (HE) staining. XZ and WC conducted data analysis. YL, Y-FC and QX wrote the final version of the manuscript. All authors contributed to the article and approved the submitted version.

## Funding

This work was supported by the Basic and Applied Basic Research of Guangdong Province (2019B1515210034, 2019A1515012006), Key Research and Development Program of Guangdong Province (2020B020222001), Chief expert Project of Agricultural Industry Technology system in Guangdong Province (2021KJ128, 2020KJ128).

## Conflict of interest

The authors declare that the research was conducted in the absence of any commercial or financial relationships that could be construed as a potential conflict of interest.

## Publisher’s note

All claims expressed in this article are solely those of the authors and do not necessarily represent those of their affiliated organizations, or those of the publisher, the editors and the reviewers. Any product that may be evaluated in this article, or claim that may be made by its manufacturer, is not guaranteed or endorsed by the publisher.

## Supplementary material

Supplementary Figure 1 Phylogenetic tree based on VacJ **(A)**, PlpE **(B)** and OmpH **(C)** sequences among strains of P. multocida.

Supplementary Figure 2 DNA identity matrix based on pairwise comparison of VacJ**(A)**, PlpE**(B)** and OmpH**(C)** sequences among strains of P. multocida.
