# Corrigendum: Immunogenicity and protective efficacy of the recombinant *Pasteurella multocida* lipoproteins VacJ and PlpE, and outer membrane protein H from P. multocida A:1 in ducks

**DOI:** 10.3389/fimmu.2022.1128242

**Published:** 2023-01-05

**Authors:** Yajuan Li, Junfang Xiao, Yung-Fu Chang, Hui Zhang, Yutao Teng, Wencheng Lin, Hongxin Li, Weiguo Chen, Xinheng Zhang, Qingmei Xie

**Affiliations:** ^1^ Heyuan Branch, Guangdong Provincial Laboratory of Lingnan Modern Agricultural Science and Technology, College of Animal Science, South China Agricultural University, Guangzhou, China; ^2^ Guangdong Engineering Research Center for Vector Vaccine of Animal Virus, College of Animal Science, South China Agricultural University, Guangzhou, China; ^3^ College of Veterinary Medicine, Cornell University, Ithaca, NY, United States; ^4^ South China Collaborative Innovation Center for Poultry Disease Control and Product Safety, College of Animal Science, South China Agricultural University, Guangzhou, China

**Keywords:** *Pasteurella multocida*, outer membrane protein, lipoprotein, immunogenicity, protective efficacy

In the published article, there was an error in the article title. Instead of “*Immunogenicity and protective efficacy of the recombinant Pastureland multocida lipoproteins VacJ and PlpE, and outer membrane protein H from P. multocida A:1 in ducks*”, it should be “*Immunogenicity and protective efficacy of the recombinant Pasteurella multocida lipoproteins VacJ and PlpE, and outer membrane protein H from P. multocida A:1 in ducks*”.

The authors apologize for this error and state that this does not change the scientific conclusions of the article in any way. The original article has been updated.

