# Supplementary material for: Immunogenicity and protective efficacy of the recombinant Pasteurella multocida lipoproteins VacJ and PlpE, and outer membrane protein H from P. multocida A:1 in ducks
Source: Front Immunol. 2022 Oct 7;13:985993. doi: 10.3389/fimmu.2022.985993 (PMC9585203; doi:10.3389/fimmu.2022.985993)
Supplement: Supplementary file 1 [file DataSheet_1.doc]

Table s1. PCR primers for recombinant target gene

| Primers name | Primer sequence（5’-3’） | Amplimer size (bp) |
| --- | --- | --- |
| rVacJ | F: caccgccaactggtctggtcccccggggcagcat  gatcgatagtaagaccgg | 746 |
|  | R: gaatgaaattgattaagcttgcggccgcacagctgt  atacacgtgcaagcca |  |
| rPlpE | F:caccgccaactggtctggtcccccggggcagcatgt  gcagtggtggtggtg | 1021 |
|  | R: ggcttgcacgtgtatacagctgtgcggccgcaagc  ttactgtgcctgatgg |  |
| rOmpH | F:accgccaactggtctggtcccccggggcagcatg  gcaaccgtgtataatca | 1083 |
|  | R: cacgtgtatacagctgtgcggccgcaagcttaatgg  tgatgatgatgatg |  |

Table S2. VacJ gene sequence used

| Isolates | Serotypes | Host species | Gene bank  accession number |
| --- | --- | --- | --- |
| IndPm167 |  | Duck | JX184900 |
| IndPm224 | A:1 | Rabbit | KJ191750 |
| IndPm176 | A:1 | Chicken | JX184901 |
| IndPm115 | B:2 | Buffalo | KJ191748 |
| IndPm111 | D:1 | Swine | KJ191746 |
| P52 | B:2 | Cattle | JX184899 |
| IndPm113 | A:1 | Sheep | KJ191747 |
| IndPm94 | A:1 | Quail | KJ191745 |
| IndPm116 | A:1 | Goat | KJ191749 |
| IndPm219 | A:1 | Turkey | JX184902 |

Table S3. PlpE gene sequence used

| Isolates | Serotypes | Host species | Gene bank  accession number |
| --- | --- | --- | --- |
| X-73 | A:1 | Chicken | EF219452 |
| P-1059 | A:3 | Turkey | EF219455 |
| P-61 | D:3 | Swine | EF219454 |
| BNM-P52 | B:2 | Cattle | GQ202239 |
| ATCC12948 | D:11 |  | EF219457 |
| P-1662 | A:4 |  | EF219456 |
| HNA32 | A | Swine | MF706321 |
| BNM-47 | D:1 |  | GQ202241 |
| HND1 | D | Swine | MF706296 |
| HNA10 | A | Swine | MF706304 |
| P407 | A:3 | Swine | EF219453 |
| C48-1 | A:5 |  | GU108958 |
| HNA44 |  | Swine | MK028802 |
| HNA40 |  | Swine | MK028801 |
| HNW14 |  | Swine | MF706308 |
| HND9 | D | Swine | MF706303 |
| HND2 | D | Swine | MF706297 |
| HNF39 |  | Swine | MK028800 |
| HNF36 |  | Swine | MK028798 |
| BNM-194 | A:3 |  | GQ202240 |

Table S4. OmpH gene sequence used

| Isolates | Serotypes | Host species | Gene bank  accession number |
| --- | --- | --- | --- |
| X-73 | A:1 | Chicken | U50907 |
| CHN-2 | D:3 | Swine | HM486501 |
| P52 | B:2 | Cattle | EU016232 |
| XJNKY-12-YF1 | A | Sheep | JX473022 |
| XJ121 | A | Cattle | JX473019 |
| CSWRIAHPmD16 | D | Sheep | MH764611 |
| P-1662 | A:4 | Turkey | U52201 |
| HND1 | D | Swine | MK028754 |
| ATCC:15743 | A:3 |  | GQ914772 |
| IndNIVEDIPm40 | A | Swine | MW142233 |
| P1059 | 3 | Turkey | U52200 |
| XJNKY-10-2YF1-2 | A | Sheep | JX473021 |
| 3397A |  |  | EF635423 |
| CSWRIAHPmA16 | A | Sheep | MH764608 |
| Pm-439 |  |  | DQ417891 |
| XJ149 |  | Cattle | JQ082509 |
| Pm-001 |  |  | DQ417873 |
| HNF39 |  | Swine | MK028782 |
| HNF36 |  | Swine | MK028779 |
| HNA32 |  | Swine | MK028775 |
| HNW14 |  | Swine | MK028764 |
| HND9 |  | Swine | MK028760 |
| HND2 |  | Swine | MK028755 |
