# Supplementary figures and images for: Immunogenicity and protective efficacy of the recombinant Pasteurella multocida lipoproteins VacJ and PlpE, and outer membrane protein H from P. multocida A:1 in ducks
Source: Front Immunol. 2022 Oct 7;13:985993. doi: 10.3389/fimmu.2022.985993 (PMC9585203; doi:10.3389/fimmu.2022.985993)

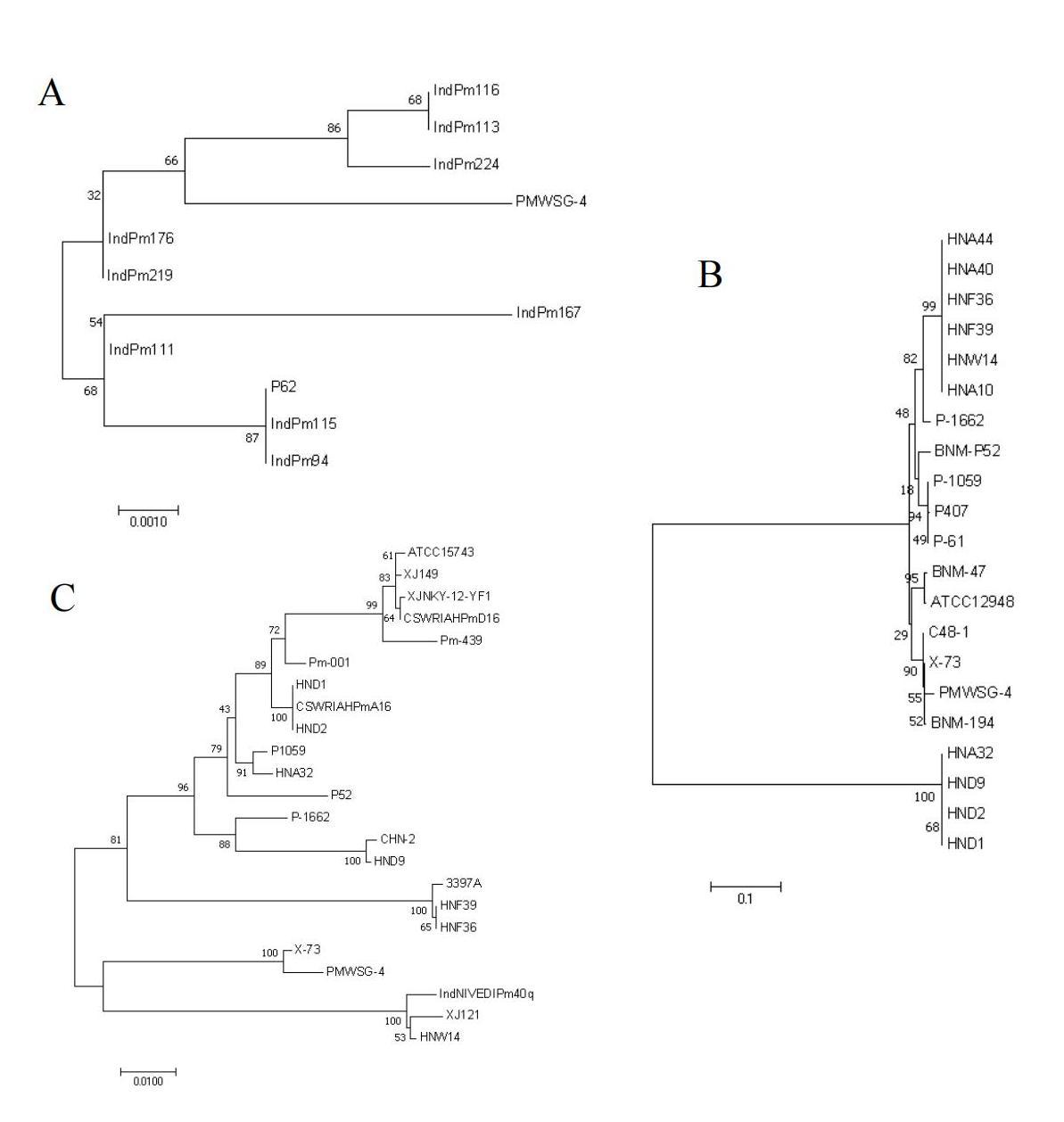

Supplement: Supplementary Figure 1 — Phylogenetic tree based on VacJ (A), PlpE (B) and OmpH (C) sequences among strains of P. multocida. [file Image_1.jpeg]

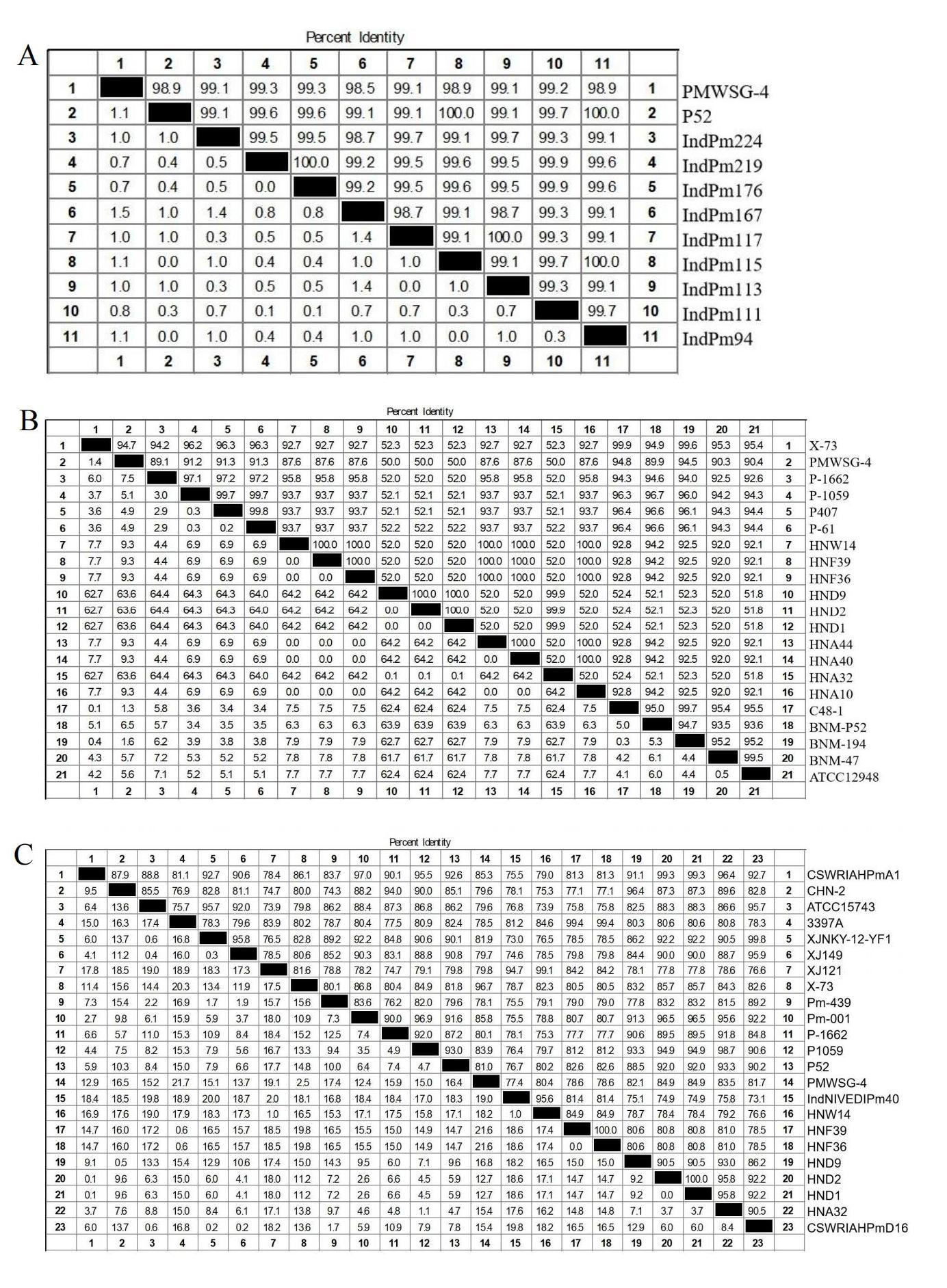

Supplement: Supplementary Figure 2 — DNA identity matrix based on pairwise comparison of VacJ(A), PlpE(B) and OmpH(C) sequences among strains of P. multocida. [file Image_2.jpeg]
